# Oral cavity cancer trends over the past 25 years in Hong Kong: a multidirectional statistical analysis

**DOI:** 10.1186/s12903-015-0074-y

**Published:** 2015-07-24

**Authors:** Keisuke Ushida, Colman P McGrath, Edward C M Lo, Roger A Zwahlen

**Affiliations:** Discipline of Oral and Maxillofacial Surgery, Faculty of Dentistry, The University of Hong Kong, 34 Hospital Road, Sai Ying Pun, Hong Kong, SAR PR-China; Discipline of Dental Public Health, Faculty of Dentistry, The University of Hong Kong, 34 Hospital Road, Sai Ying Pun, Hong Kong, SAR PR-China

**Keywords:** Oral cavity cancer, Epidemiology, Age, period, and cohort analysis, Joinpoint regression analysis

## Abstract

**Background:**

Even though oral cavity cancer (OCC; ICD 10 codes C01, C02, C03, C04, C05, and C06) ranks eleventh among the world’s most common cancers, accounting for approximately 2 % of all cancers, a trend analysis of OCC in Hong Kong is lacking. Hong Kong has experienced rapid economic growth with socio-cultural and environmental change after the Second World War. This together with the collected data in the cancer registry provides interesting ground for an epidemiological study on the influence of socio-cultural and environmental factors on OCC etiology.

**Methods:**

A multidirectional statistical analysis of the OCC trends over the past 25 years was performed using the databases of the Hong Kong Cancer Registry. The age, period, and cohort (APC) modeling was applied to determine age, period, and cohort effects on OCC development. Joinpoint regression analysis was used to find secular trend changes of both age-standardized and age-specific incidence rates.

**Results:**

The APC model detected that OCC development in men was mainly dominated by the age effect, whereas in women an increasing linear period effect together with an age effect became evident. The joinpoint regression analysis showed a general downward trend of age-standardized incidence rates of OCC for men during the entire investigated period, whereas women demonstrated a significant upward trend from 2001 onwards.

**Conclusions:**

The results suggest that OCC incidence in Hong Kong appears to be associated with cumulative risk behaviors of the population, despite considerable socio-cultural and environmental changes after the Second World War.

## Background

Even though oral cavity cancer (OCC) ranks eleven among the world’s most common cancers, accounting for approximately 2 % of all cancers [1], adequate trend analysis of it is lacking in Hong Kong. Its world’s age-standardized incidence rates (ASR) per 100'000 population are 5.3 for men and 2.5 for women [2]. There is great regional discrepancy in OCC incidence rates. In countries such as Sri Lanka, India, Pakistan and Bangladesh, OCC represents the most common cancer in men [3]. Other high-risk regions include Southeast Asia, Eastern Europe, and parts of South America. Recently increasing OCC incidence among adults below 45 years of age has been detected in Western countries [4], and the reason for this is still heatedly debated [5, 6]. Whereas tobacco and excessive alcohol consumption have been considered to be the major risk factors of OCC [7, 8], other factors like human papillomavirus infection, nutrition and socioeconomic situation seem to contribute to an increased OCC incidence [3, 9, 10].

Information about OCC has been gathered in Hong Kong and entered into the Cancer Registry since as early as 1963. Specific data on OCC related to specific oral sites according to the ICD 10 classification [11] C01 to C06, have been routinely collected. The ASR of OCC in Hong Kong is 3.9 for men and 2.4 for women [12]. Even though these figures represent a low or medium OCC risk for the overall population, the incidence rates in Hong Kong show annual variations, probably due to complex time trends. Such complex trends may reflect in part Hong Kong’s economic and immigration situations. Rapid economic growth after the Second World War (WWII) along with continuous immigration of people mainly from Southeastern Chinese Provinces, such as Guangdong and Fujian have lead to considerable changes in living and health conditions in Hong Kong. Both of these circumstances may influence the trend of OCC incidents rates. This study aimed to describe and analyze OCC development trends in Hong Kong over the last 25 years, and to study the roles of socio-environmental factors related to OCC.

## Methods

### Source of data

The Hong Kong Cancer Registry is an official member of the International Association of Cancer Registries [13]. Detailed data of cancer incidence and mortality are documented in its official website covering the time frame from 1983 to 2012 [12]. The following cancers were included into the study as OCC: cancers of base of tongue (ICD 10: C01), other and unspecified parts of tongue (C02), gum (C03), floor of mouth (C04), palate (C05), and other and unspecified parts of mouth (C06).

### Statistical analysis

The ASRs of each gender for each year between 1986 and 2010 were calculated by using the direct method for age-adjusting, according to the World Health Organization World Standard Population 2000. Cases over 20 years of age were categorized into three age groups by gender: Group 1: 20 to 44 years of age, Group 2: 45 to 64 years of age, and Group 3: aged 65 or above. For each age group, the annual age-specific incidence rates between 1986 and 2010 were calculated. Groupings and calculation of age-standardized incidence rates and age-specific incidence rates were performed directly in the website of the Hong Kong Cancer Registry with its own Cancer Statistics Query System [12].

To estimate the secular trends of OCC incidence in Hong Kong, the annual percentage change of incidence rates was calculated for the entire period of observation, 1986 to 2010, both for each age group and also the entire population of each gender. The annual percentage change estimation was based on the assumption that the incidence rates changed constantly and linearly on the log-scale [14]. Besides, a joinpoint analysis was conducted to investigate the chronological point in time of the trend change in a linear slope [15]. In this joinpoint analysis, significant changing points of the incidence trends and annual percentage change values for each segmented trend line were computed. For all calculations, a P-value of less than 0.05 was considered to be statistically significant. All calculations of the annual percentage change and joinpoint analyses were performed with the ‘Joinpoint’ software version 3.5.4 provided by the US National Cancer Institute on a Windows XP Home Edition (National Cancer Institue, Bethesda, MD).

An age, period, and cohort (APC) model was used to analyze the influences of chronological age, population’s birth cohort, and time period of the diagnosis [16]. With respect to the chronological age, the population was divided into ten groups; nine quinquennial age groups from 40 to 84 years of age, and a group of 85 years of age or above. Regarding the time period of diagnosis, the entire 25 year observation period from 1986 to 2010 was split into five intervals, each encompassing a period of 5 years. Fourteen overlapping birth cohort periods were set between 1896 and 1965 at 10 year intervals, starting with the cohort from 1896 to 1905, ending with the one from 1961 to 1970. Related to the assumption that OCC incidence rates follow the Poisson distribution, seven statistical models in log-linear scale were established containing the following effects; age-only (A), period-only (P), cohort-only (C), age-period (AP), age-cohort (AC), and full APC. Akaike’s Information Criteria (AIC) of each model was confirmed to estimate their reliability of the models.

Due to linear dependences between age, period and cohort effects (Cohort = Period - Age), it is well understood that the concomitant estimation of these three effects is impossible. This fundamental “identification problem” of the APC model estimation was tackled with the following mathematical measures:

1. AIC calculation of A, P, and C models.
2. As the P model demonstrated the least goodness of fit depicting nearly a simple linear function for both genders, an alternative P model was calculated, including fewer parameters and a better goodness of fit. The P model where P was replaced by 1) a constant (*y = a, a is constant*) was named “P-constant” model; 2) a linear function (*y = ax + b, a and b are constants*) was termed “P-linear” model; and 3) a quadratic function (*y = ax^2 + bx + c, a,b and c are constants*) was called “P-quadratic” model.
3. AICs of the above three alternative period models were calculated to find out the best fitted alternative model for each gender group.
4. Better matching period models were extrapolated to AP and APC models in order to achieve better fitting and to overcome the “identification problem” of a full APC models.

All calculations related to age, period, and cohort analysis were performed on Macintosh OSX version 10.8.1 with ‘JMP 10 (for Macintosh)’ software version 10.0.0. (SAS Institute Inc., Cary, NC) [17].

## Results

### Age-standardized and age-specific incidence rates

**Fig. 1:**
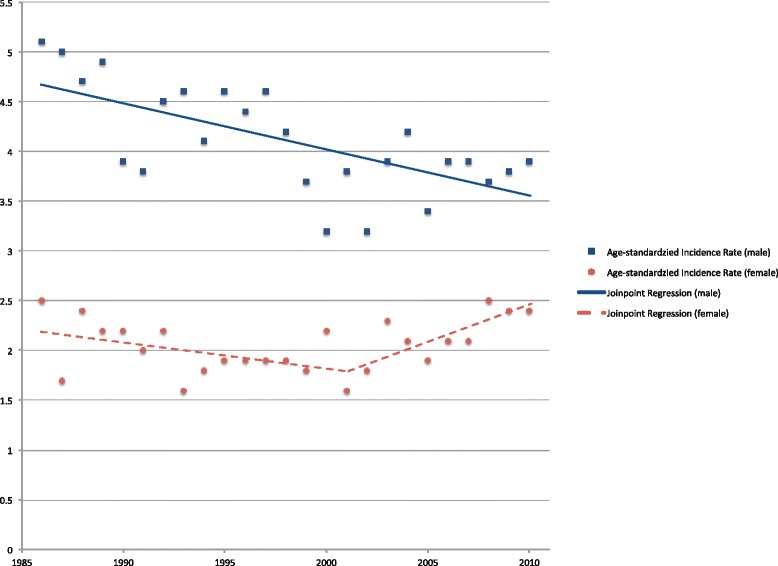
Age-standardized incidence rates of OCC (C01-C06) in Hong Kong. Age-standardized incidence rates of OCC per 100,000 persons in Hong Kong and the results of joinpoint regression analyses from 1986 to 2010

ASRs for OCC in Hong Kong, from 1986 to 2010, are highlighted in Fig. 1. Of an overall 5888 cases, 29 (0.5 %) cases were 20 years old or younger. They were excluded from age-specific incidence rates calculation, as no OCC case was reported in this age group for each gender in half of the period years. Men showed a continuous downward trend during the entire period with an annual percentage change of −1.2 (95 % C.I.: −1.7, −0.6). No significant trend change was found in the joinpoint regression analysis. On the other hand, the trend in women showed a significant change in the year 2001, joinpoint in 2001 (95 % C.I.: 1991, 2006). Compared with the initially gentle downward trend with an annual percentage change of −1.2 (95 % C.I.: −2.6, 0.2), the second part of the graph shows an increased upward trend with an annual percentage change of 3.5 (95 % C.I.: 0.5, 6.7).

**Fig. 2:**
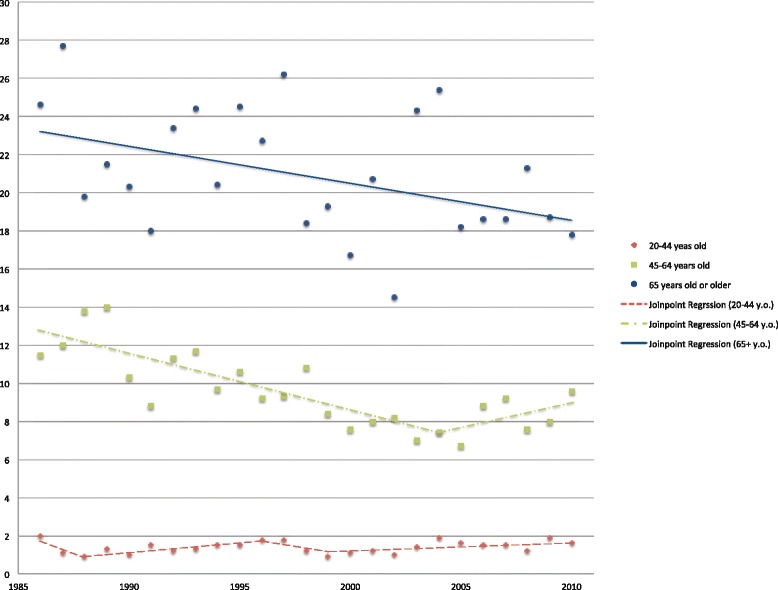
Age-specific incidence rates of OCC (C01-C06) in Hong Kong among men. Age-specific incidence rates of OCC per 100,000 persons in Hong Kong among men, and the results of joinpoint regression analyses from 1986 to 2010

**Fig. 3:**
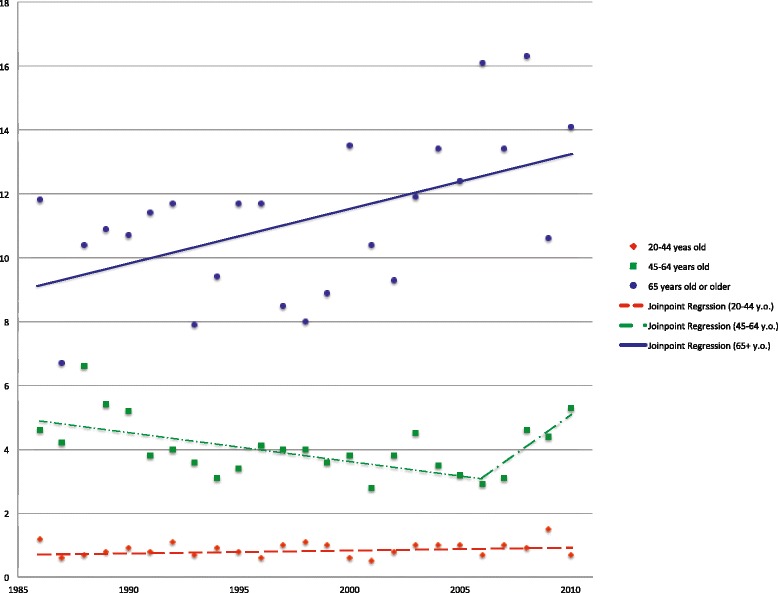
Age-specific incidence rates of OCC (C01-C06) in Hong Kong among women. Age-specific incidence rates of OCC per 100,000 persons in Hong Kong among women, and the results of joinpoint regression analyses from 1986 to 2010

Age-specific incidence rates of the middle age groups, 45 to 64 years of age, are highlighted in Figs. 2 and 3 (Fig. 2 for men, Fig. 3 for women). Both genders showed significant trend changes around the year 2005, the jointpoint for men is in 2004 (95 % C.I.: 1999, 2007) while that for women is in 2006 (95 % C.I.: 2003, 2008). The first downward trend in the annual percentage change value in both gender groups were statistically significant: for men: −3.1 (95 % C.I.: −4.1, −2.0), for women: −2.2 (95 % C.I.: −3.5, −0.9). In the second part of the graphs, women showed a steeper rise of incidence rates with an annual percentage change of 13.7 (95 % C.I.: −2.3, 32.5), compared to men. Age-specific incidence rates in old age groups, 65 years old or older, and the results of the joinpoint regression analyses are also shown in Figs. 2 and 3. Whereas incidence rates among men are in a gradual decline during the entire period with an annual percentage change of −0.9 (95 % C.I.: −1.7, −0.1), rates among women show a significant upward trend with an annual percentage change of 1.6 (95 % C.I.: 0.5, 2.7).

### Age, period, and cohort model

**Fig. 4:**
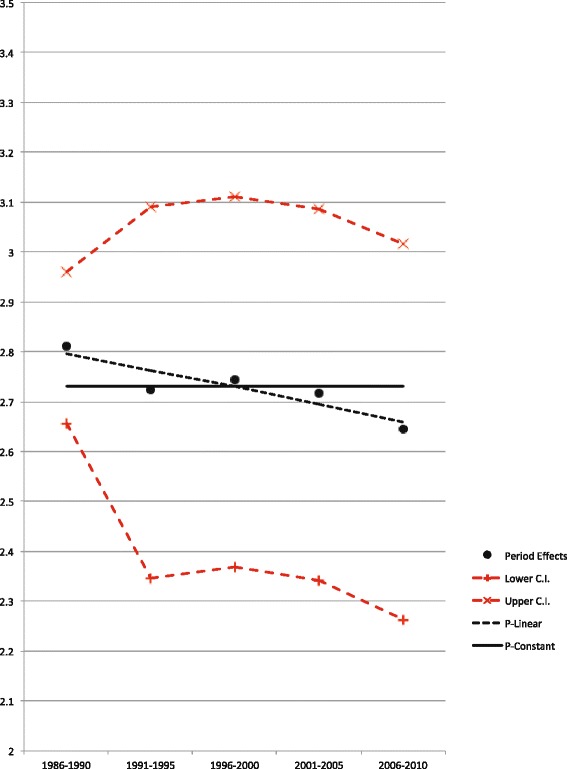
Estimated period and alternative period models (relative risks) among men. Estimated period and alternative period models (relative risks), and the 95 % confidence interval (C.I.) of oral cancer incidence rates among men, from 1986 to 2010. A continuous line represents the best fit model

**Fig. 5:**
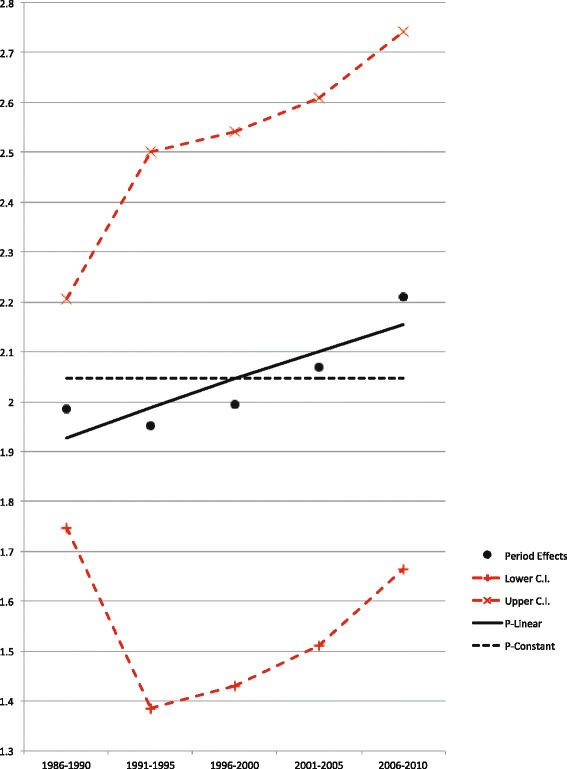
Estimated period and alternative period models (relative risks) among women. Estimated period and alternative period models (relative risks), and the 95 % confidence interval (C.I.) of oral cancer incidence rates among women, from 1986 to 2010. A continuous line represents the best fit model

The goodness of fit for different models using age, period and cohort effects was evaluated by calculating AIC. The smaller the AIC value, the better the model fit is. Because the P model showed the least goodness of fit, a constant, and a linear and quadratic function within each period parameter were extrapolated to find a model with better goodness of fit. For men, the “P-constant” model showed a better fit compared to the original “P”, “P-linear”, and “P-quadratic” models, whereas for women the “P-linear” model showed a better fit (Fig. 4 for men, Fig. 5 for women, Table 1). AICs of modified models according to above results are shown in Table 2. An “AP (P-linear)” model, for example, means that a “P-linear” model was extrapolated to an AP model, whereas an “APC (P-constant)” model is the same as an AC model. An “AP (P-constant)” model, same as an A model, is the best fit model for men (AIC = 269.8), whereas an “AP (P-linear)” model suited best for women (AIC = 223.8).

**Table 1:** Different fits of the models of period (P) and alternative P models

Gender	Model	Number of parameters	d.f.	AIC
Men	P	5	45	464.5
	P-constant	1	49	458.7
	P-linear	2	48	458.9
	P-quadratic	3	47	460.9
Women	P	5	45	340.7
	P-constant	1	49	336.2
	P-linear	2	48	335.6
	P-quadratic	3	47	336.7

Different fits of the models of period (P) and alternative P models of OCC incidence rates (C01-C06) in Hong Kong, from 1986 to 2010. (d.f.: degree of freedom, AIC: Akaike’s Information Criteria, smaller number shows the better fitting)

**Table 2:** Summary of different fits of modified models

Gender	Model	Number of parameters	d.f.	AIC
Men	A	10	40	269.8
	P-constant	1	49	458.7
	C	14	36	294.1
	AP	14	36	275.6
	AP (P-constant)	10	40	269.8
	AC	23	27	285.1
	APC (P-constant)	23	27	285.1
Women	A	10	40	224.3
	P-linear	2	48	335.6
	C	14	36	259.9
	AP	14	36	228.9
	AP (P-linear)	11	39	223.8
	AC	23	27	244.5
	APC (P-linear)	24	27	246.5

Summary of different fits of age (A), best-fitted period (P), cohort (C), age and period (AP), age and cohort (AC), and age, period and cohort (APC) model of OCC incidence rates (C01-C06), from 1986 to 2010. Models whose P effects are extrapolated by the best-fitted P models; hence in men, the AC model equals the APC (P-constant) model. (d.f.: degree of freedom, AIC: Akaike’s Information Criteria)

**Fig. 6:**
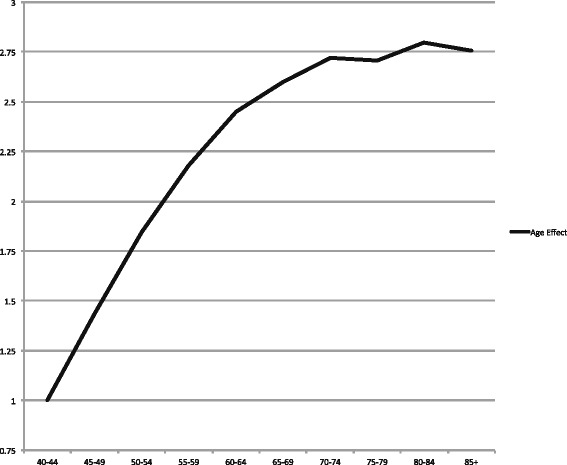
Estimated age effects (relative risks) from the best fitting model among men. Estimated age effects (relative risks) from the Age and P-Constant model of OCC incidence rates (C01-C06) among men, from 1986 to 2010

**Fig. 7:**
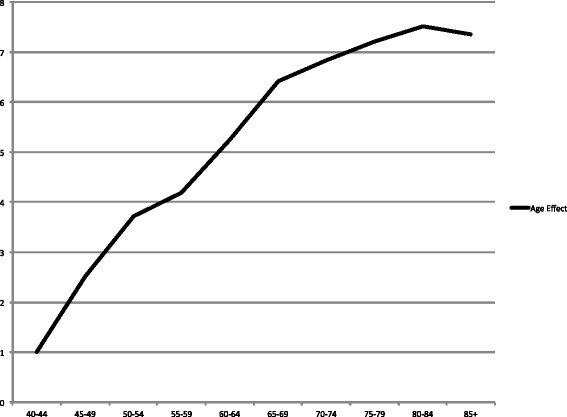
Estimated age effects (relative risks) from the best fitting model among women. Estimated age effects (relative risks) from Age and P-Linear model of OCC incidence rates (C01-C06) among women, from1986 to 2010

Figure 6 displays the estimated relative risks (RRs) of the “AP (P-constant)” model for men, while the estimated RRs for women are highlighted in the “AP (P-linear)” model (Fig. 7). In both genders, the risk for OCC increases steadily with age.

## Discussion

Using modified multidirectional statistical approaches, this study found that people's risk behaviors are decisive for the OCC incidence in Hong Kong. After the WWII, Hong Kong had experienced rapid population growth due to immigration and also substantial economic development. Within 50 years, its population and the gross domestic product per capita had almost decupled. Since its handover in 1997, Hong Kong has retained its former city-state character as a special administrative region in China. Due to this confined geopolitical position, Hong Kong is able to set up an almost complete database on cancer cases, population, economy and public health data. As previously described, the data credibility of the Hong Kong Cancer Registry has undergone substantial improvement after 1983 [18]. Liability to register each case of death with its particular cause of death together with a steady decrease in cases without a death certificate helped raising the proportion of verified cancer cases to 99.3 % in 2009 [19]. Furthermore, both native inhabitants and immigrants were mostly from Southeastern Chinese provinces, hence of similar genetic background. For above reasons, Hong Kong might have an ideal population to shape and test hypotheses about cancer risks including socio-environmental factors.

In this study, APC modeling detected that age effect together with a linearly increasing period effect were the most probable explanations for OCC development rates in women in Hong Kong. While age effect is a common etiological factor for many diseases and aspects of daily life, period effect points at existing potential age-independent factors influencing subjects [16]. In Hong Kong men, it seems that a constant period together with an age effect determines the OCC rates. In both gender groups, the age effects of the model sway more with increasing age, being in line with reports from other countries [3]. It therefore might be assumed that OCC development in Hong Kong is also mainly caused by cumulative exposure to risk factors. While the cohort effect may be associated directly with exposure on the day of birth, it also might represent a quantification of the generational lifestyle changes after birth [20]. Assuming that cohort effects are of less importance in OCC development, the main causative factor in Hong Kong might be the age factor together with tobacco smoking, which have been found in other places [9, 21, 22].

Although the APC modeling is employed to understand the time-dependent changes of age, period, and cohort effects individually, its calculation and interpretation are attended with much complexity due to an inherent “identification problem”. To compensate for this difficulty, joinpoint regression analysis of both age-standardized and age-specific incidence rates was performed. In line with high-risk countries such as India and Sri-Lanka [23, 24], as well as with many European countries [9] and Northern China [25], the OCC incidence rates for Hong Kong men were decreasing during the period from 1986 to 2010. On the contrary, an evident upward trend during the last 5 to 10 years was detected in Hong Kong women. Of special interest are middle aged men, where the incidence rate has been rising since 2004. This rise, however, does not reach a statistically significant level of the annual percentage change value. This is probably due to the rather short observational period subsequent to a significant change confirmed by the joinpoint regression analysis. In the last few decades, long-term upward trends for OCC incidence have been detected mainly among women in economically developed countries [26]. A similar change in trend can be detected in Hong Kong women, starting from 2000. Specifically, middle aged women reveal an obvious upward trend, with a particular joinpoint in 2006.

Nonetheless, there are shortcomings of this study. First, the lack of a pathological tumor staging makes investigations on improvements in OCC diagnosis, screening procedures, consultation rates and their implications on mortality rates over time impossible. The fact that Hong Kong residents born prior to 1960s often had no birth certificates to verify their age represents a second shortcoming, which, however, might have been outweighed in this study with the APC modeling. Another severe limitation is the lack of information concerning alcohol consumption in Hong Kong. Alcohol is considered to be a major risk factor, accounting for 7 - 19 % of OCC [21]. Only since 2004, detailed data of alcohol consumption have been collected in Hong Kong as a part of the "Behavioral Risk Factor Surveillance System". This unfortunately prevents any interpretation and statistical analyses regarding chronological changes and eventual effects on OCC development. Future epidemiological research needs to consider the specific biological association between dominant risk habits and OCC development, a strategy which might provide insight into the particular pathogenesis of OCC in Hong Kong. As is well known, smoking is regarded as the main dose-dependent risk of OCC [8, 21, 22]. Approximately one-quarter of OCC cases are assumed to have causal association with smoking [21]. The Hong Kong Cancer Registry has recently started to collect some basic demographic data, such as topography and morphology of the cancer, while information regarding smoking habits of each case is still lacking [19]. Thus, it is not possible to discuss in detail the association between smoking and OCC development in Hong Kong.

## Conclusion

OCC development in Hong Kong can be considered to be mainly subject to cumulative exposure to risk factors over time, but not substantially to geo-political or socio-geographical factors related to its socio-cultural melting-pot character.

## Abbreviations

A: Age-only
AC: Age-cohort
AIC: Akaike’s Information Criteria
AP: Age-period
APC: Age, period, and cohort
ASR: Age-standardized incidence rate
C: Cohort-only
OCC: Oral cavity cancer
P: Period-only
RR: Relative risk
WWII: Second World War
